# Intoxication from an Accidentally Ingested Lead Shot Retained in the Gastrointestinal Tract

**DOI:** 10.1289/ehp.7594

**Published:** 2005-02-10

**Authors:** Per Gustavsson, Lars Gerhardsson

**Affiliations:** ^1^Department of Occupational and Environmental Health, Stockholm Centre for Public Health, Stockholm, Sweden; ^2^Division of Occupational Health, Department of Public Health Sciences, Karolinska Institutet, Stockholm, Sweden; ^3^Department of Occupational and Environmental Medicine, Sahlgrenska University Hospital, Göteborg, Sweden

**Keywords:** blood lead, diagnosis, gastrointestinal tract, health effects, lead shot

## Abstract

A 45-year-old woman was referred to the Department of Occupational and Environmental Health in January 2002 because of increased blood lead concentrations of unknown origin. She suffered from malaise, fatigue, and diffuse gastrointestinal symptoms. She had a blood lead level of 550 μg/L (normal range < 40 μg/L). The patient had not been occupationally exposed to lead, and no potential lead sources, such as food products or lead-glazed pottery, could be identified. Her food habits were normal, but she did consume game occasionally. Clinical examination, including standard neurologic examination, was normal. No anemia was present. Laboratory tests showed an increased excretion of lead in the urine, but there were no signs of microproteinuria. An abdominal X ray in October 2002 revealed a 6-mm rounded metal object in the colon ascendens. Before the object could be further localized, the patient contracted winter vomiting disease (gastroenteritis) and the metal object was spontaneously released from the colon during a diarrhea attack. The object was a lead shot pellet, possibly but not normally used in Sweden for hunting wild boar or roe deer. Blood lead levels slowly decreased. Nine months later the patient’s blood lead levels were almost normal (~ 70 μg/L) and her symptoms had almost completely disappeared. In this case, a rare source of lead exposure was found. In investigations of blood lead elevations of unknown origin, we recommend abdominal X ray in parallel with repeated blood lead determinations.

A 45-year-old woman who had suffered from gastrointestinal (GI) symptoms similar to irritable bowel disease since adolescence sought a private practitioner in 1991 when she suspected medical problems from amalgam dental fillings. In addition to the bowel symptoms, she suffered from fatigue. An analysis of the metal content in the patient’s feces showed considerably increased concentrations of mercury, cadmium, and lead. In 1992 she was referred to the Department of Occupational and Environmental Medicine of Huddinge Hospital, Stockholm, Sweden, for further investigation. No source of occupational or environmental metal exposure was identified, and the patient showed blood concentrations of mercury and cadmium within normal ranges. The patient’s blood lead concentration was 100 μg/L. The reference level used by the analytical laboratory at that time was < 145 μg/L. The analysis of metals in feces is considered much more unreliable than levels in blood, and the physician concluded that there was no evidence of environmental exposure to lead, mercury, or cadmium. Chelation therapy with dimercaptosuccinic acid (DMSA), which had been initiated by the practitioner in 1991, was continued for 2 years. The patient received oral treatment two to three times per week, but we do not know the exact dose. Symptoms were mainly unchanged during the treatment period.

In August 2001 when the patient saw another physician, a moderately increased blood lead level of 210 μg/L was found (normal range in unexposed subjects < 40 μg/L). At that time, the DMSA medication was started again, and she was referred to the Department of Occupational and Environmental Health in Stockholm. A repeated blood lead sample in December 2001 showed an even higher blood lead concentration of 550 μg/L.

This patient was born in Germany in 1956 and moved to Sweden in the mid-1970s. During 1980–1994 she gave birth to eight children, the last of them twins. In the early 1980s she worked at day care centers, and in 1997 she began working part-time cleaning buildings. The family lived in a house built in the 1930s. She was a smoker during the 1980s (except during pregnancy), but she quit smoking in the early 1990s. Her alcohol consumption was low, about one bottle of wine per month, and she did not abuse drugs. She had no psychiatric problems.

During the investigation at the Department of Occupational and Environmental Health she reported increasing GI problems with daily diarrhea for about a year. She also suffered from coldlike symptoms in combination with malaise and fatigue several times a week. A clinical examination, including a standard neurologic examination (standard arm and leg reflexes, skin sensibility, and two-point discrimination in hands) was normal.

Figure 1 shows the development of the patient’s blood lead pattern. Blood lead levels peaked in December 2001, and thereafter a gradual decline was evident. Beginning in December 2001, all analyses were performed by inductively coupled plasma-mass spectrometry at laboratories that were accredited for analysis of lead in blood; previous samples (from August 2001 and 1991) were not analyzed at accredited laboratories. The DMSA treatment that had been started in 2001 was discontinued in February 2002.

In January 2002, we began our investigation by asking the patient about potential lead sources in her diet or in the environment. She had no contact with lead crystal glassware or lead-glazed pottery, and her food habits were normal. The blood lead concentrations in the other family members were normal. Her hematologic parameters and kidney function were normal, and she showed no signs of microproteinuria. In October 2002, lead in urine was increased (75 μg/L; reference value < 30 μg/L), and an X ray of the abdomen showed a dense rounded metal object with a diameter of approximately 6 mm at the colon ascendens. While waiting for a computed tomography (CT) scan, which we planned in order to localize the object more precisely, the patient contracted the winter vomiting disease (gastroenteritis) in January 2003. During severe diarrhea, the object was released from the GI tract. The object was identified as lead shot pellet used for game hunting, and marks on it showed that it had been fired through a rifle. The lead shot pellet had a diameter of 6 mm and a mass of 1.7 g (Figure 2). A new abdominal X ray confirmed that the object was no longer in the colon.

The woman confirmed that she had consumed game at several occasions: she had eaten wild boar at a restaurant in Sweden in 1993, and hare or rabbit on some occasions during the 1990s, both in Sweden and in Germany. However, she could not recall having eaten meat that contained a hard object at any time. Her blood lead levels in April 2003, 2 months after the elimination of the lead shot pellet from her colon, were still high (345 μg/L). After another 7 months, the patient’s blood lead concentration was 72 μg/L, almost down to reference levels. At that time, the attacks of malaise and fatigue had disappeared, and the abdominal symptoms were mild. Since 2003 she has been working full-time.

## Discussion

Lead intoxication may be caused by intake of food and water containing increased lead concentrations or by industrial exposure from inhalation of lead-contaminated air. The absorption of ingested lead varies from 10 to 60% (Tsuchiya 1986), with an average absorption of stable lead of 15–20% in adults (Skerfving 1993). It is less common for lead objects to be swallowed and retained in the GI tract. However, similar reports have been found in the literature. Laszloffy et al. (1998) reported a 17-year-old male who accidentally swallowed a fishing weight; the weight was retained in the stomach and caused severe lead poisoning, including disturbance of liver function and signs of encephalopathy. Because it was impossible to remove the foreign object by gastroscopy, a gastrotomy was performed, followed by intravenous treatment with a chelating agent; the result of this treatment was a complete clinical recovery.

Durlach et al. (1986) described the case of a 30-year-old farmer, a frequent hunter and consumer of game, who was admitted to an emergency unit because of severe abdominal pain. Urography was undertaken, showing 29 lead pellets scattered in the areas of the appendix and adjacent parts of the colon. High lead levels were found in his blood (674 μg/L) and urine. After treatment with enema and EDTA salt infusions, his condition improved. X rays, however, revealed that 14 lead pellets were still trapped in the appendix. Accordingly, an appendectomy was performed to cure the patient from his lead poisoning. Thereafter, the blood lead concentration gradually returned to normal.

Madsen et al. (1988) studied patients prospectively referred to a hospital for routine radiography of the abdomen. Seven patients with one or two lead shot pellets retained in the appendix were identified. For each patient, two age- and sex-matched controls without lead shot pellets in the appendix were selected. None of the patients with lead shot pellets had blood lead levels near toxic levels, but nevertheless their median blood lead levels were almost twice as high (114 μg/L vs. 60 μg/L) compared with the controls.

**Figure 1:**
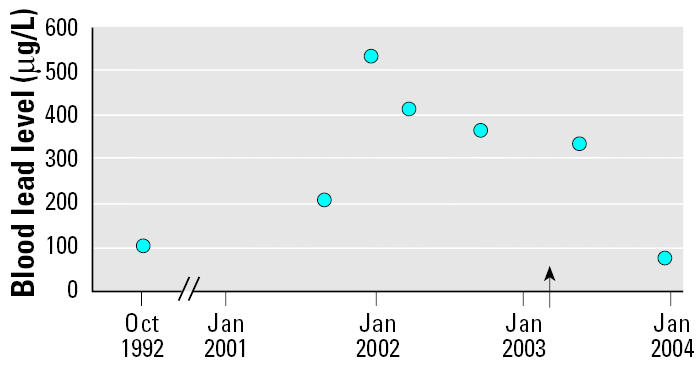
Patient’s blood lead concentrations from 1992 to 2003. The arrow indicates the elimination of the lead shot pellet.

Although our patient’s blood lead level of 100 μg/L in 1992 was within the reference range of the analytical laboratory, it was somewhat higher than would be expected among unexposed individuals (at that time < 60 μg/L). We do not know the reason for this, but because the patient’s blood lead level was only slightly above the normal range in 1992, it is probable that the intake of the lead shot pellet took place between 1993 and 2001. The rapid increase in blood lead level from August to December of 2001 indicates that the lead shot pellet may have been ingested in the autumn of that year. The lead shot pellet was released from the GI tract in January 2003. Thereafter, a slow decline in blood lead level took place, and the blood lead level was still clearly elevated in April 2003 (345 μg/L). A blood lead sample 7 months later (November 2003) showed an almost normal blood lead level (74 μg/L; Figure 1). It seems clear that the high blood levels were caused by the lead shot, even if it is not possible to determine exactly when it was ingested.

The lead shot pellet was larger (diameter 6 mm) than those normally used for hunting in Sweden and was a type not allowed in the country. It may have been used for hunting wild boar or roe deer in Germany and may also have been used rarely in Sweden (Hagberg S, personal communication).

The blood lead levels that we observed, if they resulted from long-term exposure, could be associated with GI disturbances (which the patient had) and neurophysiologic findings of impaired nerve transmission. However, anemia or other symptoms of lead intoxication would not be expected. The patient promptly recovered from the malaise, and her bowel problems gradually decreased after the elimination of the lead shot pellet. It is likely that the bowel symptoms were caused by the lead exposure, but because the patient suffered from bowel problems earlier in life, we cannot be certain that the two are linked.

Similar blood lead patterns have been observed for other individuals with retained lead objects in the GI tract. An 8-year-old boy swallowed 20–25 fishing sinkers and a nail (Mowad et al. 1998). He quickly reached a blood lead concentration of 540 μg/L. He recovered after whole-bowel irrigation, colonoscopy, and oral succimer treatment.

Because children have a considerably higher lead absorption in the GI tract (30–40%) than adults (15–20%), it is especially important to promptly examine and diagnose children with suspected lead objects retained in the GI tract. Also, necessary treatment should not be delayed. Otherwise, children may reach toxic blood lead levels in a few days. Even fatal lead encephalopathy could be caused by heavy exposure (Hugelmeyer et al. 1988).

For diagnosis, we recommend venous sampling for blood lead determination in combination with X-ray screening, especially in children with a medical history of pica behavior (Mowad et al. 1998) or after suspected ingestion of lead bodies. An alternative to X-ray investigations is examination with a metal detector, which seems to be a simple and accurate technique to identify ingested metal objects of various types (Arena and Baker 1990; Tidey et al. 1996). Identification of sources of elevated blood lead levels by isotope-specific analyses of ^207^Pb, ^206^Pb, and ^204^Pb in environmental and biological samples has been suggested and applied for children (Gwiazda and Smith 2000).

Pica behavior during pregnancy has been reported as a cause of blood lead elevations both in mother and child (Hamilton et al. 2001). However, our patient was not pregnant during 1991–1992 or during the present investigation in 2001–2003.

Historically, lead acetate has been used as a sweetener and for antibacterial purposes in wine (e,g., during the Roman Empire). Some centuries ago it was discovered that crystal glass with high concentrations of lead shows a high durability and brilliance. The lead concentration in crystal glassware is often 25–30%; it has been shown that wine kept in lead crystal glass containers for a long time may contain a considerable amount of lead. Graziano and Blum (1991) performed an experiment in which port wine containing 89 μg/L lead was kept in lead crystal decanters. After 4 months, the lead level in the wine reached 3.5 mg/L; long-term storage yielded concentrations exceeding 20 mg/L. Other experiments with wine glasses showed that lead began to elute from lead crystal within minutes (Graziano and Blum 1991).

## Conclusions

Our investigation revealed an unusual source of lead exposure in this patient. In the first phase, we directed the investigation toward finding an environmental source, most probably a lead-containing food product. Accidental intake of a lead shot pellet was not suspected, and we have found very few earlier reports of this type of exposure among adults.

Lead objects retained in the GI tract must be diagnosed and treated promptly. This is especially important in children who have a considerably higher GI absorption than adults and who can reach toxic levels of blood lead within a couple of days. We suggest an X ray of the abdomen in cases where external sources of lead exposure have been eliminated. The patient should also be followed by regular blood lead determinations. If possible, lead objects could be removed from the GI tract by gastroscopy or colonoscopy. Indications for and choice of chelation therapy depend not only on blood lead levels but also on factors such as severity of symptoms, age of the patient, and exposure circumstances (Dietrich et al. 2004; Landrigan 1994).

## Figures and Tables

**Figure 2:**
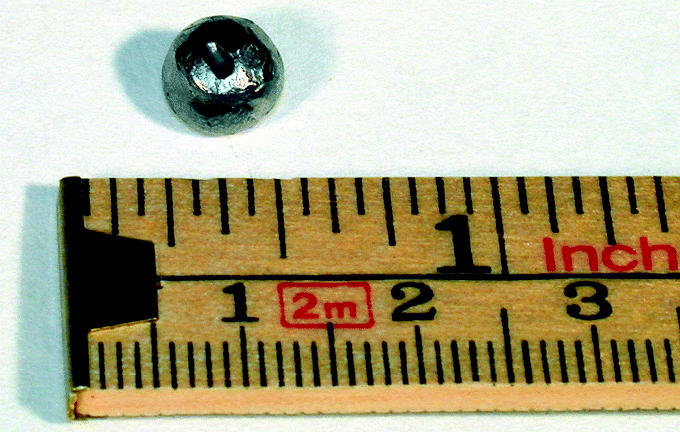
Photograph of the lead shot pellet. Photographed by G. Hagelthorn.
